# Utility of Lymphocyte Transformation Test for Assisting Updated Roussel Uclaf Causality Assessment Method in Drug-Induced Liver Injury: A Case-Control Study

**DOI:** 10.3389/fphar.2022.819589

**Published:** 2022-03-10

**Authors:** Amelia Rodríguez, Irene García-García, Lucía Martínez de Soto, Arturo Gómez López De Las Huertas, Alberto M. Borobia, Andrea González-Torbay, Ibtissam Akatbach-Bousaid, Miguel González-Muñoz, Elena Ramírez

**Affiliations:** ^1^ Clinical Pharmacology Department, Faculty of Medicine, La Paz University Hospital-IdiPAZ, Universidad Autónoma de Madrid, Madrid, Spain; ^2^ Immunology Department, La Paz University Hospital-IdiPAZ, Madrid, Spain

**Keywords:** drug-induced liver injury (DILI), clinical pharmacology, pharmacovigilance, updated roussel uclaf causality assessment method (updated RUCAM), causality assessment, lymphocyte transformation test (LTT), adverse drug reaction (ADR)

## Abstract

**Background:** The Roussel Uclaf Causality Assessment Method (RUCAM) is a validated tool for assessing causality in cases of suspected drug-induced liver injury (DILI). However, RUCAM cannot discriminate between concomitant hepatotoxic drugs with the same temporal sequence.

**Objective:** To analyse the utility of the lymphocyte transformation test (LTT) for assisting updated RUCAM in 45 patients and 40 controls with a clinical diagnosis of DILI.

**Methods:** Suspected DILI cases were detected through the Prospective Pharmacovigilance Program from Laboratory Signals in Hospital (PPLSH) or by consultations. The controls completed the drug therapy with no adverse reactions during the study period. A receiver operating characteristics (ROC) curve analysis was performed to calculate the optimal cut-off value for the stimulation index (SI), corresponding to the largest sum for the specificity and sensitivity values of LTT for true DILI cases.

**Results:** Out of 45 patients diagnosed with DILI, 42 cases were detected by the PPLSH, two cases by consultation and one case by both methods. Most DILI cases (64.4%) arose during hospitalization. According to the biochemical parameters, 24 cases (53.3%) had the hepatocellular phenotype, 14 (31.1%) had the cholestatic phenotype, and 7 cases (15.6%) had the mixed phenotype. Considering the severity criteria, 7 (15.5%) cases were classified as moderate DILI, and 4 (8.9%) were severe DILI; there were no fatal cases. A total of 149 drugs (median/case, 3; IQR, 2–5) were suspected to be involved in the DILI cases (RUCAM score ≥3). In 8 cases, only one drug was suspected, and polypharmacy (≥5 drugs) was identified in 29% of the cases. Of all DILI cases, 46 (30.9%) of the 149 suspected drugs produced positive LTT results, and the LTT was positive in 34 (75.5%) of the 45 patients. No exposed controls produced positive LTT results. The optimal cut-off of 1.95 for the SI was obtained with a sensitivity of 77% and specificity of 100% (area under the curve, 0.91; 95% asymptotic confidence interval 0.84–0.97; *p* < 0.001). The sensitivity of the hepatocellular phenotype was 92%.

**Conclusion:** Our results demonstrate that LTT is an add on strengthening causality in cases of suspected idiosyncratic DILI, especially for patients with several suspected drugs and a hepatocellular phenotype.

## Introduction

Drug-induced liver injury (DILI) is an uncommon but potentially lethal adverse drug reaction, with an annual incidence ranging from 2.4 to 23.8 per 100,000 patients. DILI is the most frequent cause of acute liver failure in North America and Europe, the main reason for drugs failing to obtain marketing authorisation, and a frequent cause for post-marketing restrictions and withdrawal of products. DILI can mimic almost any known type of liver disease, making the differential diagnosis challenging. DILI reactions are commonly categorised as intrinsic (i.e., predictable following excess drug exposure), idiosyncratic (rare but potentially severe due to unique host susceptibility factors), or indirect (unintended injuries due to a drug’s biological actions) (Andrade et al., 2019).

The diagnosis of DILI is complicated when it is a dose-independent or idiosyncratic adverse reaction (Andrade et al., 2019), especially when the patient has been exposed to more than one drug (García-Cortés et al., 2011; Weber et al., 2021). The diagnosis is based on ruling out other potential causes for the liver disorders and on applying causality algorithms to the suspected drugs when patients have been exposed to several drugs.

Causality algorithms are structured and standardised scales for quantifying the association between the drug and the DILI. Currently, the Roussel Uclaf Causality Assessment Method (RUCAM) is the only clinically validated liver-specific scale and the most widely used algorithm, not only for individual cases of DILI but also for prospective and retrospective studies (Danan and Teschke, 2019). The algorithm consists of 7 criteria: 1) time to DILI onset from when the drug was ingested, 2) rate of improvement with drug cessation, 3) risk factors for DILI, 4) potential influence of concomitant drugs, 5) exclusion of alternative causes for the liver disorders, 6) the drug’s known hepatotoxicity and 7) response to unintentional re-exposure. RUCAM ranks DILI as highly probable, probable, possible, unlikely or excluded.

RUCAM is easy to use, decreases the interindividual and intraindividual variability and increases the reproducibility (Danan and Teschke, 2019). However, the RUCAM scale has the limitation that when hepatotoxic drugs are ingested at approximately the same time, an accurate causality assessment becomes difficult, even when alternative causes have been ruled out (García-Cortés et al., 2011; Weber et al., 2021).

T-cell sensitisation to a drug can be studied using the Lymphocyte Transformation Test (LTT), which is based on the activation and expansion of the drug-specific memory T cells after an *in vitro* incubation of the patient’s peripheral mononuclear cells with different concentrations of the suspected drugs (Pichler and Tilch, 2004). The LTT has been employed to improve the diagnosis of DILI cases in a study of 95 patients diagnosed with DILI, in which a positive LTT to at least one of the suspected drugs was obtained in 56% of the cases and, no reactivity was found in the controls (Maria and Victorino, 1997).

In an attempt to improve the diagnosis of DILI and identify the culprit drug(s), the aim of this study was to analyse the utility of combining RUCAM and LTT for 45 patients with a clinical diagnosis of DILI, as well as 40 tolerant controls.

## Materials and Methods

### Setting and Patients

La Paz University Hospital in Madrid, Spain, is a tertiary-care teaching facility, where from 2007 all admissions to wards have been monitored by a Pharmacovigilance Program from Laboratory Signals in Hospital (PPLSH) in order to proactively detect serious adverse drug reactions. (Ramirez et al., 2010). A retrospective, case-control study, using DILI detected from the PPLSH or from consultations of other specialties, was conducted between 2019 and 2021 in the Pharmacovigilance Unit of the Clinical Pharmacology Department of La Paz University Hospital (Madrid, Spain). The study was approved by the La Paz University Hospital Ethics Committee (Code PI-3226; 25 May 2018). Due to the retrospective nature of the study, the absence of informed consent was permitted. For all patients initially categorised as having suspected DILI, a complete report was submitted to the pharmacovigilance centre in Madrid, Spain (https://www.notificaram.es).

Forty-five suspected DILI cases were consecutive detected through the PHPLS programme (Ramirez et al., 2010) or by consultations and were referred to Pharmacovigilance Unit for follow-up.

The inclusion criteria for the DILI cases were 1) the DILI criteria were met (Aithal et al., 2011), 2) all alternative causes of RUCAM (Danan and Teschke, 2016) (groups I and II) were reasonably ruled out, and 3) at least one drug had a RUCAM rank ≥3. Poisonings and medication errors were excluded from the study.

Age- and sex-matched patients who completed the drug therapy with no adverse reactions during the study period were assigned as tolerant controls.

### Case Detection, Definition and Severity Criteria

The procedure of PPLSH for detecting DILI cases has been described elsewhere. (Ramirez et al., 2010). Briefly, in phase I, on-file laboratory data at admission or during hospitalisation were screened 7 days a week, 24 h a day, for results of alanine aminotransferase (ALT) ≥5 times the upper limit of normal (ULN). In phase II, the patients were identified to avoid duplicates, and electronic medical records were reviewed. In those cases where ALT was clearly attributable to other alternative causes, the patients were not further analyzed because an DILI was unlikely. In phase III, a case-by-case evaluation was performed for the remaining cases. When the drug history was unclear, we interviewed the patients or their relatives to obtain more details and conducted additional tests. Once ruled out alternative causes for DILI, the suspicious drugs were withdrawn after discussion with the attending physician and patients were offered to be followed in the Pharmacovigilance Unit.

The case definition of DILI relied on the following clinical chemistry criteria (Aithal et al., 2011): 1) ALT levels ≥5 times the ULN, 2) alkaline phosphatase (ALP) levels ≥2 times the ULN or 3) ALT levels ≥3 times the ULN and, simultaneously, bilirubin levels >2 times the ULN.

DILI severity was defined according to the following criteria (Aithal et al., 2011): 1) mild: ALT/ALP levels meeting the DILI criteria but with bilirubin levels <2 times the ULN; 2) moderate, ALT/ALP levels meeting the DILI criteria and bilirubin concentration ≥2 times the ULN or symptomatic hepatitis; 3) severe, ALT/ALP levels meeting the DILI criteria and bilirubin concentrations ≥2 times the ULN and one of the following: 1) international normalised ratio (INR) ≥1.5, 2) ascites and/or encephalopathy, disease duration <26 weeks, and absence of underlying cirrhosis, and 3) other organ failure considered to be due to DILI; and 4) fatal outcome or transplantation.

### Phenotype Characterisation of DILI

A DILI episode can be characterised as hepatocellular, mixed or cholestatic, based on the ratio (R) value defined as the ratio of serum ALT to ALP elevations [expressed as ULN multiples (× ULN)] at the onset of DILI. Hepatocellular, cholestatic and mixed episodes of DILI tend to have different outcomes and recovery rates. Determining the R value is recommended for all patients with suspected DILI to help categorise the type and pattern of liver injury (Danan and Teschke, 2016). The classification employed to differentiate DILI cases detected by the PHPLS (Aithal et al., 2011) was the following:

- Hepatocellular: R ≥ 5
- Cholestatic: R ≤ 2- Mixed: 2 < R < 5

### Causality Assessment

The causality assessment was performed using updated RUCAM, the most commonly employed algorithm for assessing causality in DILI (Danan and Teschke, 2016; Teschke, 2019; Teschke and Danan, 2020). The updated RUCAM algorithm evaluates the possibility that a drug is responsible for the DILI, scoring according to 7 separate domains related to the temporal relationship between exposure to a drug and the liver injury (both its onset and course), the exclusion of an alternative non-drug-related cause, exposure to other drugs that could also explain DILI, patient risk factors for the adverse hepatic event, evidence in the literature regarding the relationship between the drug and the event, and the effect of the drug in the event of re-exposure. The total score (ranging from −7 to +14) from the domain-specific assessment classifies the drug into 5 separate categories: highly probable (≥9), probable (6–8), possible (3–5), unlikely (1–2) or excluded (<0). A RUCAM score ≥3 was considered drug-related.

### *In vitro* Lymphocyte Transformation Test

LTT was performed using different concentrations of the drug(s) involved in the DILI cases (RUCAM score ≥3) and tolerant controls. LTT was performed after DILI recovery and at least 1 month after steroid therapy was stopped, if applicable. Lymphocyte proliferation was measured as previously described (Pichler and Tilch, 2004; Vílchez-Sánchez et al., 2020). Mononuclear cells were separated over a density gradient (Histopaque-1077, Sigma-Aldrich) from fresh peripheral blood and were plated in flat bottom wells of microtitre plates at 2 × 10^5^ cells/well. Cells were incubated for 6 days with various drug concentrations in triplicate. Drugs were assayed at concentrations of 1, 10 and 100 μg/ml, and occasionally, a lower or higher concentration (0.1, 200 or 500 µg) were used, as previously described (Pichler and Tilch, 2004). We used phytohemagglutinin (5 μg/ml) as a positive control. For the final 18 h of the incubation period, proliferation was determined by adding 1 µCi [^3^H] thymidine. Proliferative responses were calculated as the stimulation index (SI), defined as the ratio between the mean values of the counts per minute in cultures with drug and those obtained without drug. The threshold for positivity was assessed by receiver-operating characteristics (ROC) analysis. For this purpose, LTT was analysed in 40 participants tolerant to the drugs assayed in the patient group. An LTT result was considered positive when the SI exceeded the threshold in at least one drug concentration. Patients were considered as having immune-mediated DILI when at least one LTT was positive.

### Statistical Analysis

By accepting an alpha risk of 0.05 and a beta risk of 0.2 in a two-sided test, using arcsin-approximation we needed 41 participants for the first group and 41 for the second to determine a statistically significant proportion difference, which we expected to be 0.74 for group 1 and 0.95 for group 2. Continuous variables are expressed as mean and standard deviation (SD) or median and interquartile range (IQR), according to the Kolmogorov-Smirnov normality test. Categorical variables are expressed in absolute terms and percentages. We employed the chi-squared test to compare the categorical variables and employed Student’s t-test for the continuous variables with a normal distribution. In the event the data did not have a normal distribution, we used the nonparametric Mann-Whitney *U*-test or Kruskal Wallis test, as appropriate. Differences were considered significant when the *p*-value was <0.05.

We performed a receiver operating characteristics (ROC) curve analysis to calculate the optimal cut-off value for SI, corresponding to the largest sum for the specificity and sensitivity values, between cases with a clinical diagnosis of DILI and tolerant controls. A sensitivity analysis was performed with different RUCAM scores. The data were analysed using the IBM SPSS Statistics version 21.0 (IBM Corporation, Armonk, NY, United States).

## Results

### Characteristic of DILI Cases

Out of 45 patients diagnosed with DILI, 42 cases were detected by the PPLSH, two cases by consultation and one case by both methods. Most DILI cases (64.4%) arose during hospitalization (Table 1). The patients’ mean age (standard deviation, SD) was 49.9 (±19.6) years, and 26 were women (57.8%). There were no statistically significant differences compared with the controls’ mean age (42.1 ± 20.8 years; *p* = 0.077) and sex distribution (29 women; *p* = 0.156). The median maximum disturbance in the liver function test during a DILI episode was 358 IU/L (IQR, 255–732) for ALT, 150 IU/L (IQR, 97–286) for ALP, 232 IU/L (IQR, 146–674) for AST and 0.7 mg/dl (IQR, 0.4–1.9) for bilirubin. According to the biochemical parameters, 24 cases (53.3%) had the hepatocellular phenotype, 14 (31.1%) had the cholestatic phenotype, and 7 cases (15.6%) had the mixed type. Considering the severity criteria, 7 (15.5%) cases were classified as moderate DILI, and 4 (8.9%) were classified as severe DILI. Cases with moderate DILI presented symptomatic hepatitis (fatigue, nausea, vomiting, jaundice), while severe cases showed coagulopathy (International normalised ratio >1.5). There were no fatal cases. Except for one moderate case that occurred during hospitalization, the rest of the moderate or severe cases caused hospitalization (10/11, 90.9%). On the other hand, of the 34 mild DILI cases, 28 (82.4%) occurred during hospitalization. The median latency (time from drug intake to alterations in the liver function test) for all patients, for those with the hepatocellular phenotype, and for the cholestatic/mixed cases was 6 days (IQR, 4–16), 6 days (IQR, 4–18) and 7 days (IQR, 3–15), respectively. Latency was significantly longer in DILI cases resulting in hospitalization, 12 days (IQR 9–18), compared to DILI cases that occurred during hospitalization, 6 days (IQR 3–8) (*p* = 0,04). The median interval between the onset of a reaction and the study for all patients, for those with the hepatocellular phenotype, and for the cholestatic/mixed cases was 7 months (IQR, 5–8), 7 months (IQR, 6–10) and 6 months (IQR, 4–8), respectively. There were no statistically significant differences between the hepatocellular and the cholestatic/mixed cases in terms of latency (*p* = 0.706) or time to the study (*p* = 0.072).

**TABLE 1:** Patients’ clinical and demographic characteristics.

case	Age, years	Sex	Liver injury	ALT (IU/L) (ULN = 35)	ALP (IU/L) (ULN = 116)	AST (IU/L) (ULN = 40)	Bilirubin (mg/dl) (ULN = 1.2)	DILI severity (symptoms)
1	86	M	Cholestatic	416	720	379	1.6	Moderate (fatigue, jaundice)
2	62	M	Cholestatic	328	568		0.6	Mild
3	59	M	Cholestatic	308	652	240	1.9	Mild
4	67	F	Hepatocellular	1,679	271	792	2.1	Moderate (fatigue, nausea, vomiting)
5	56	F	Hepatocellular	202	101	170	0.4	Mild
6	88	M	Hepatocellular	1,010	108	783	0.8	Mild
7	23	F	Hepatocellular	536	47	1,297	1.09	Mild
8	69	F	Cholestatic	417	325	223	0.7	Mild
9	57	F	Hepatocellular	175	85	148	0.7	Mild
10	38	F	Hepatocellular	2,208	156	1,612	3.3	Severe (jaundice, coagulopathy)
11	33	F	Hepatocellular	1,401	100		2.3	Moderate (jaundice)
12	14	F	Hepatocellular	2013	261	2,785	3.5	Severe (fatigue, nausea, vomiting, coagulopathy)
13	58	M	Cholestatic	261	163	143	0.4	Mild
14	2	F	Hepatocellular	2039	549	2,308	6.9	Moderate (jaundice)
15	31	M	Mixed	924	315	1,494	0.7	Mild
16	45	F	Cholestatic	192	362	117	0.5	Mild
17	62	M	Cholestatic	154	742	277	1.3	Mild
18	83	F	Cholestatic	362	379	563	0.9	Mild
19	59	F	Hepatocellular	475	116	143	0.5	Mild
20	13	F	Cholestatic	418	231		0.6	Mild
21	58	F	Cholestatic	358	201	225	0.4	Mild
22	49	M	Hepatocellular	541	75		0.5	Mild
23	69	F	Cholestatic	319	302	190	4.7	Moderate (jaundice)
24	37	M	Mixed	342	151	272	0.6	Mild
25	40	M	Mixed	245	95	133	0.4	Mild
26	49	F	Hepatocellular	177	77	150	0.5	Mild
27	46	M	Hepatocellular	183	72	80	0.6	Mild
28	44	F	Mixed	510	217	520	1.9	Moderate (fatigue, nausea, vomiting)
29	29	F	Cholestatic	279	159	118	0.4	Mild
30	54	M	Hepatocellular	249	49	192	0.7	Mild
31	68	M	Hepatocellular	345	83	225	0.2	Mild
32	42	M	Hepatocellular	2,555	144		16.9	Severe (jaundice, coagulopathy)
33	51	M	Hepatocellular	216	88	84	0.8	Mild
34	33	F	Hepatocellular	4,091	111	2,596	16.6	Severe (jaundice, coagulopathy)
35	32	F	Mixed	305	150	242	0.2	Mild
36	78	F	Mixed	260	126	97	1.0	Mild
37	45	M	Hepatocellular	508	70	375	0.6	Mild
38	55	F	Cholestatic	285	173		0.8	Mild
39	44	M	Mixed	481	127	183	0.4	Mild
40	41	M	Hepatocellular	1,269	146	638	0.5	Mild
41	39	F	Mixed	311	129	111	0.4	Mild
42	60	F	Hepatocellular	3,014	164	3,125	2.2	Moderate (jaundice)
43	26	F	Cholestatic	436	396		0.3	Mild
44	80	M	Hepatocellular	228	49	172	0.4	Mild
45	73	F	Hepatocellular	251	99	240	0.3	Mild

Abbreviations, DILI; drug-induced liver injury, ALP; alkaline phosphatase, ALT; alanine aminotransferase, AST, aspartate aminotransferase; F, female; M, male; ULN, upper limit of normal.

### Culprit Drugs

A total of 149 drugs (median/case, 3; IQR 2–5) were suspected to be involved in DILI (Table 2). In 8 cases, only one drug was suspected, and polypharmacy (≥5 drugs) was identified in 29% of the cases. All drugs were ranked ≥3 by RUCAM. Sixty-one drugs were scored as possible, 81 as probable, and 7 as highly probable. Patients were not re-challenged with suspected drugs; however, case 7 tolerated the three suspected drugs after accidental re-exposure.

**TABLE 2:** Drugs involved in drug-induced liver injury.

case	Drugs	RUCAM score	LTT (SI)	No. of drugs	No. of positive LTTs
1	Fosfomycin	7	1.30	2	0
	Ciprofloxacin	9	1.80		
2	Ceftriaxone	5	1.40	2	1
	Piperacillin/Tazobactam	5	2.30		
3	Acetylsalicylic acid	5	0.80	6	2
	Amoxicillin	6	0.60		
	Azathioprine	7	0.80		
	Clarithromycin	6	1.10		
	Metamizole	4	2.00		
	Nitroglycerine	4	2.40		
4	Cefditoren	5	4.10	3	2
	Ezetimibe	8	3.50		
	Atorvastatin	4	1.10		
5	Amoxicillin	7	3.00	3	1
	Acetaminophen	7	0.60		
	Metamizole	6	0.70		
6	Amoxicillin	10	16.50	2	1
	Levofloxacin	5	1.00		
7	Amoxicillin	7	1.50	3	0
	Ibuprofen	7	1.00		
	Acetaminophen	7	1.00		
8	Omeprazole	7	0.40	4	1
	Escitalopram	7	3.70		
	Ciprofloxacin	7	0.40		
	Amoxicillin	7	0.40		
9	Clomipramine	9	2.30	4	2
	Trazodone	6	1.60		
	Venlafaxine	10	4.00		
	Vortioxetine	7	0.50		
10	Amoxicillin	6	1.00	9	2
	Cefazolin	6	1.00		
	Dexketoprofen	6	0.80		
	Indomethacin	6	1.20		
	Ibuprofen	5	0.70		
	Lidocaine	3	0.70		
	Mepivacaine	4	2.00		
	Metamizole	6	0.80		
	Acetaminophen	5	0.90		
11	Labetalol	7	0.40	3	2
	Amlodipine	4	2.80		
	Methyldopa	6	3.00		
12	Pyrazinamide	6	4.20	2	1
	Rifampicin	3	1.30		
13	Pyrazinamide	8	5.10	2	2
	Rifampicin	3	2.50		
14	Isoniazid	9	3.20	1	1
15	Amoxicillin	6	1.20	3	0
	Ibuprofen	7	0.40		
	Acetaminophen	7	0.60		
16	Dexketoprofen	6	1.20	5	1
	Levofloxacin	6	1.50		
	Amikacin	4	2.20		
	Metamizole	4	1.502		
	Acetaminophen	4	1.603		
17	Levofloxacin	7	1.100	5	0
	Piperacillin/Tazobactam	7	1.200		
	Clarithromycin	6	1.101		
	Ceftriaxone	6	1.301		
	Dexketoprofen	6	1.100		
18	Cefazolin	7	0.901	5	2
	Quetiapine	7	4.301		
	Tramadol	6	1.502		
	Sulfamethoxazole	6	5.80		
	Ferrous gluconate	6	1.70		
19	Diazepam	3	30.30	4	3
	Acetaminophen	3	2.50		
	Dexketoprofen	3	6.00		
	Sulpiride	3	1.30		
20	Meropenem	4	1.30	1	0
21	Acetaminophen	5	1.70	2	0
	Ibuprofen	5	1.20		
22	Praziquantel	6	1.30	5	1
	Rifampicin	6	1.30		
	Cloxacillin	6	1.00		
	Cefepime	6	0.60		
	Vancomycin	5	2.60		
23	Amoxicillin	8	11.20	2	1
	Ezetimibe	4	1.50		
24	Amoxicillin	3	1.30	3	0
	Piperacillin/Tazobactam	3	0.60		
	Levetiracetam	3	0.60		
25	Lopinavir/ritonavir	4	3.20	1	1
26	Azithromycin	7	1.60	6	1
	Tocilizumab	7	1.30		
	Hydroxychloroquine	7	1.60		
	Lorazepam	5	2.40		
	Acetaminophen	4	1.10		
	Metamizole	4	2.00		
27	Hydroxychloroquine	7	3.00	6	2
	Ceftriaxone	6	1.80		
	Azithromycin	7	1.80		
	Lopinavir/ritonavir	7	1.20		
	Pantoprazole	6	2.80		
	Ciprofloxacin	6	2.00		
28	Metamizole	6	1.40	4	1
	Tramadol	6	1.40		
	Atorvastatin	8	11.20		
	Diosmin	6	1.30		
29	Dexketoprofen	5	0.90	1	0
30	Lopinavir/ritonavir	4	1.70	5	1
	Levofloxacin	4	2.30		
	Dexketoprofen	4	1.50		
	Interferon beta	4	1.80		
	Hydroxychloroquine	4	1.90		
31	Tocilizumab	7	2.30	2	1
	Hydroxychloroquine	7	1.50		
32	Dexketoprofen	8	2.60	5	2
	Acetaminophen	5	2.201		
	Metamizole	5	1.702		
	Ibuprofen	8	1.90		
	Omeprazole	8	1.90		
33	Azithromycin	6	4.30	1	1
34	Amoxicillin	5	5.00	4	2
	Ibuprofen	5	1.90		
	Methocarbamol	5	15.80		
	Metamizole	3	0.70		
35	Ceftriaxone	6	0.70	2	0
	Acetaminophen	6	0.80		
36	Olanzapine	4	2.90	2	2
	Quetiapine	4	2.50		
37	Hydroxychloroquine	4	1.70	6	2
	Azithromycin	4	3.80		
	Dexketoprofen	4	2.80		
	Doxycycline	4	0.70		
	Omeprazole	4	1.80		
	Enoxaparin	4	1.60		
38	Atorvastatin	7	4.40	5	1
	Enoxaparin	7	0.70		
	Metamizole	6	0.70		
	Metoclopramide	4	0.50		
	Acetaminophen	8	0.90		
39	Ceftriaxone	5	2.20	1	1
40	Amoxicillin	8	1.50	5	1
	Acetaminophen	8	1.40		
	Metamizole	8	1.30		
	Enoxaparin	8	3.10		
	Omeprazole	8	1.20		
41	Acetaminophen	9	1.80	4	0
	Bemiparin	8	1.90		
	Acetylsalicylic acid	5	1.00		
	Paricalcitol	3	0.70		
42	Valaciclovir	10	1.00	3	0
	Ibuprofen	5	0.70		
	Berberine	3	0.60		
43	Ceftriaxone	5	1.80	3	1
	Dexketoprofen	5	4.00		
	Acetaminophen	3	1.70		
44	Atorvastatin	8	2.20	1	1
45	Cefuroxime	6	2.50	1	1

Abbreviations: LTT, lymphocyte transformation test; RUCAM, roussel uclaf causality assessment method; SI, stimulation index.

### LTT as a Diagnostic Tool for DILI

**FIGURE 1:**
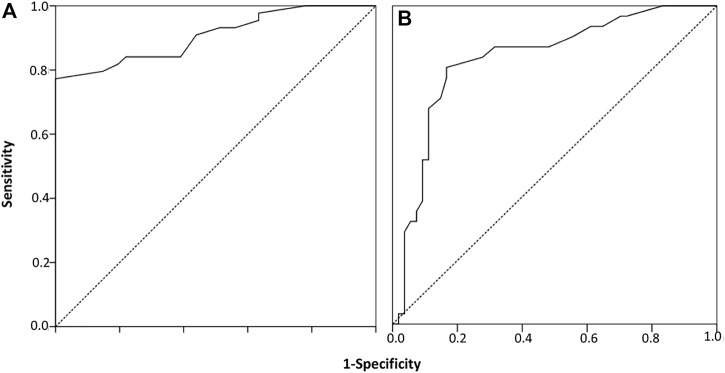
Receiver operating characteristic curve analysis for the stimulation index generated between patients (*n* = 45) and exposed controls (*n* = 40). An optimal threshold of 1.95 was obtained considering a RUCAM score ≥3 **(A)** or ≥6 **(B)** as the true positive condition for drug-induced liver injury.

**FIGURE 2:**
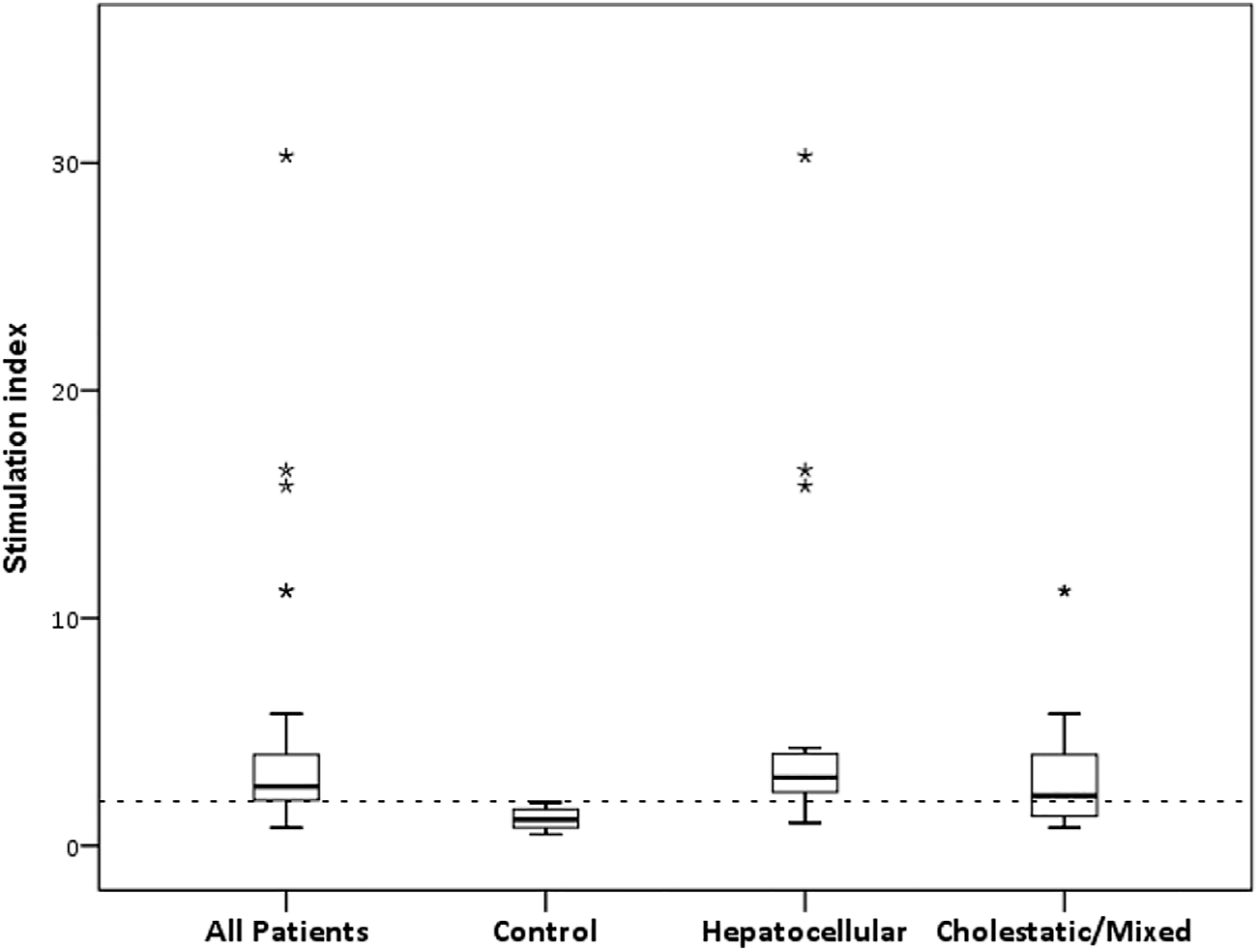
Stimulation indexes in DILI patients and tolerant controls. Box plots represent the median and IQR of SI in patients (*n* = 45), controls (*n* = 40), and hepatocellular (*n* = 24) and cholestatic/mixed (*n* = 21) phenotypes. Outliers are displayed as asterisks. Dotted line displays the threshold for positive LTT.

For LTT, we performed an ROC curve analysis to determine the best discriminative threshold for SI between the 45 cases clinically diagnosed of DILI and the 40 tolerant controls. In the cases in which different drugs were suspected, different SIs were consequently available, and the maximum SI was then considered in the analysis. To analyse how causality grading affects LTT performance, scores ≥3 and ≥6 were used to define a true positive condition for DILI, namely, a RUCAM rank of at least possible and probable, respectively (Figure 1). For ≥3, all cases were considered as true DILI, and an optimal cut-off of 1.95 for SI was obtained, with a sensitivity of 77% and specificity of 100% (area under the curve [AUC], 0.91; 95% asymptotic confidence interval [CI], 0.84–0.97; *p* < 0.001). For ≥6, 31 cases were considered as true DILI, and the optimal threshold obtained was the same, with a sensitivity of 80% and specificity of 83% (AUC, 0.83; 95% CI, 0.74–0.93; *p* < 0.001). Further analyses included all cases as true positive DILI and an SI of 1.95 as the positivity threshold. With this threshold, 46/149 (30.9%) of the suspected drugs produced a positive LTT (Table 2), and the LTT was positive for 34/45 (75.5%) patients (Figure 2), indicating a drug-specific immune response mechanism underlying their DILI. No exposed controls produced positive LTTs (Table 3).

**TABLE 3:** Lymphocyte transformation test results in tolerant controls.

Control	Age, years	Sex	Drug	LTT (SI)
1	28	Female	Acetaminophen	1.90
2	42	Female	Acetaminophen	0.50
3	88	Female	Acetaminophen	1.90
4	29	Female	Acetaminophen	1.70
5	39	Male	Acetaminophen	0.50
6	32	Female	Acetaminophen	1.00
7	42	Male	Acetaminophen	1.30
8	83	Female	Acetaminophen	0.60
9	28	Female	Ibuprofen	1.10
10	39	Male	Ibuprofen	1.60
11	38	Female	Ibuprofen	1.10
12	20	Female	Ibuprofen	1.80
13	14	Male	Ibuprofen	1.90
14	28	Female	Acetylsalicylic acid	1.80
15	35	Male	Acetylsalicylic acid	1.60
16	72	Female	Clarithromycin	1.00
17	29	Female	Dexketoprofen	1.50
18	38	Female	Dexketoprofen	1.90
19	35	Male	Dexketoprofen	1.20
20	30	Female	Dexketoprofen	1.20
21	37	Male	Dexketoprofen	0.70
22	29	Female	Amoxicillin	0.80
23	3	Female	Amoxicillin	1.90
24	40	Female	Amoxicillin	1.20
25	11	Female	Amoxicillin	1.40
26	39	Male	Metamizole	1.00
27	83	Female	Metamizole	0.70
28	67	Female	Metamizole	0.07
29	20	Female	Metamizole	0.05
30	63	Male	Metamizole	1.50
31	35	Male	Ciprofloxacin	1.60
32	43	Female	Ciprofloxacin	0.80
33	43	Female	Ciprofloxacin	0.08
34	35	Female	Cefepime	0.60
35	47	Female	Cefepime	1.90
36	38	Female	Cefazolin	0.80
37	84	Male	Omeprazole	1.30
38	51	Female	Vancomycin	0.60
39	43	Female	Levofloxacin	0.80
40	83	Female	Levofloxacin	0.80

Abbreviations: LTT, lymphocyte transformation test; SI, stimulation index

### Lymphocyte Reactivity in Relation to DILI Characteristics

To assess whether this immune response could be associated with hepatitis severity, the liver function test was compared between cases with positive LTTs (*n* = 34) and cases in which an immune response to drugs was not demonstrated (*n* = 11). No statistical differences were found in ALT, ALP, AST, and bilirubin concentrations.

Regarding the number of positive LTTs, 46.7% of the cases had only one drug inducing a positive proliferative response, 26.7% had two drugs, and 2.2% had three drugs (Table 2). To assess the putative relationship between the number of positive LTTs and liver enzyme concentrations, the liver function test was compared between cases with 1 (*n* = 21) and 2 or more (*n* = 13) positive LTTs. There was no statistical differences in the ALT, ALP, AST, and bilirubin concentrations.

The median SI was 2.6 (IQR, 1.9–4.0) for the patients and 1.1 (IQR, 0.8–1.6) for the controls (*p* < 0.001). To analyse whether different thresholds could be obtained for different phenotypes, we performed ROC curve analyses for the hepatocellular and cholestatic/mixed cases. The analysis produced a threshold of 1.95 for the hepatocellular type (*n* = 24), with a sensitivity of 92% (AUC, 0.96; 95% CI 0.91–1.00) and a threshold of 2.0 for the cholestatic/mixed phenotype (*n* = 21), with a sensitivity of 57% (AUC, 0.84; 95% CI 0.73–0.94). Therefore, an equivalent SI threshold of 2.0 could be applied to the various DILI phenotypes.

Given that more than one drug are involved in most DILI cases, we analysed the association between the number of suspected drugs and the DILI phenotype. There was no significant difference in the number of drugs involved in hepatocellular DILI (median 3, IQR 2–5) and cholestatic/mixed (median 3, IQR 2–4) DILI (*p* = 0.452). However, a higher number of positive LTTs was observed in hepatocellular DILI (median 1, IQR 1–2) than in cholestatic/mixed (median 1, IQR 0–1) DILI (*p* = 0.015). Higher SI values were found in hepatocellular DILI (median 3.0, IQR 2.3–4.0) than in cholestatic/mixed (median = 2.2, IQR = 1.3–4.2) DILI, although this difference was not statistically significant (*p* = 0.08) (Figure 2). Of note, the three cases (6, 19 and 34) with higher SIs corresponded to the hepatocellular cases.

### Lymphocyte Reactivity by Drug Subgroup

Several drug subgroups were involved in DILI (Table 4). The subgroups most frequently identified were antibiotics (*n* = 51), non-steroidal anti-inflammatory drugs (*n* = 29), analgesics (*n* = 16) and psychotropics (*n* = 12).

**TABLE 4:** Frequency of positive lymphocyte transformation test by drug group.

Drug subgroups (ATC classification)	Drug	No. of positive LTTs/No. Of cases
NSAID (M01. Anti-inflammatory and anti-rheumatic products)		
	Acetylsalicylic acid	0/2
	Metamizole	1/10
	Ibuprofen	0/7
	Dexketoprofen	4/9
	Indomethacin	0/1
Antibiotics (J01, Antibacterials for systemic use)		
	Fosfomycin	0/1
	Ciprofloxacin	0/3
	Ceftriaxone	1/6
	Amoxicillin	4/11
	Levofloxacin	1/4
	Piperacillin/Tazobactam	1/3
	Clarithromycin	0/2
	Cefazolin	0/2
	Meropenem	0/1
	Rifampicin	1/3
	Cloxacillin	0/1
	Cefepime	0/1
	Doxycycline	0/1
	Azithromycin	2/4
	Cefditoren	1/1
	Pyrazinamide	2/2
	Isoniazid	1/1
	Amikacin	1/1
	Sulfamethoxazole	1/1
	Cefuroxime	1/1
	Vancomycin	1/1
Psychotropics (N03 Antiepileptics N05 Psycholeptics N06 Psychoanaleptics)		
	Escitalopram	1/1
	Chlorimipramine	1/1
	Venlafaxine	1/1
	Quetiapine	2/2
	Diazepam	1/1
	Lorazepam	1/1
	Olanzapine	1/1
	Trazodone	0/1
	Vortioxetine	0/1
	Sulpiride	0/1
	Levetiracetam	0/1
Antihypertensives (C01, Cardiac therapy; C02, Antihypertensive; C07, Beta-blocking agents)		
	Labetalol	0/1
	Amlodipine	1/1
	Methyldopa	1/1
Lipid-lowering (C10, Agents that lower serum lipids)		
	Ezetimibe	1/2
	Atorvastatin	3/4
Anticoagulants (B01, Antithrombotic agents)		
	Enoxaparin	1/3
	Bemiparin	0/1
Antiparasitic (P02, Anthelmintics; P01, Antiprotozoal)		
	Praziquantel	0/1
	Hydroxychloroquine	1/5
Analgesics		
(N02, Analgesics)	Acetaminophen	1/14
	Tramadol	0/2
Proton pump inhibitor		
(A02BC, Proton pump inhibitors)	Omeprazole	0/4
	Pantoprazole	1/1
Others		
	Azathioprine	0/1
	Lidocaine	0/1
	Ferroglycine	0/1
	Tocilizumab	1/2
	Lopinavir/ritonavir	1/3
	Diosmin	0/1
	Interferon beta	0/1
	Metoclopramide	0/1
	Paricalcitol	0/1
	Valaciclovir	0/1
	Berberine	0/1
	Nitroglycerine	1/1
	Mepivacaine	1/1
	Methocarbamol	1/1

Abbreviations: ATC, anatomical Therapeutic Chemical (Ref. ATC/DDD Index 2021. Available online: https://www.whocc.no/atc_ddd_index/(accessed on 24 September 2021).

There were differences in the frequency of positive LTTs among the drug groups (*p* = 0.012). The groups with the highest percentage of positive LTTs were the psychotropics (8/12) and lipid-lowering (4/6) drugs.

## Discussion

The diagnosis of idiosyncratic DILI is based on ruling out other alternative causes of hepatitis, given that there is no specific biological marker supporting the DILI diagnosis available in the routine diagnostic laboratory. RUCAM can standardise and support the clinical assessment of DILI; however, the RUCAM algorithm has some limitations (Andrade et al., 2019), including that it cannot discriminate between concomitant hepatotoxic drugs with the same temporal sequence (García-Cortés et al., 2011; Weber et al., 2021). The recently published study by our group (Delgado et al., 2021) describing the characteristic of DILI cases in COVID-19 patients showed that 98% of patients had been treated with five or more drugs. The updated RUCAM scale discriminated one in five drugs (from 1,308 used drugs to 263 related drugs), 51.2% of the cases had at least one probable cause and 48.8% possible, but the median number of related drugs was 3 (range 1–7). Therefore, a tool to assist the RUCAM-based causality assessment is desirable. Identifying the culprit drug is of crucial importance for managing patients with adverse drug reactions. This identification in DILI is hampered because *in vivo* testing used in other allergic reactions either has low sensitivity, as occurs with skin testing, or is contraindicated, as in the case of re-challenge tests with suspected drugs (Phillips et al., 2019). The immune response to drugs and/or their reactive metabolites is considered one of the main pathogenic mechanisms underlying DILI. The analysis of infiltrates in liver biopsy samples from patients with DILI (Wuillemin et al., 2014) and the generation of drug-specific T-cell clones from patients with DILI indicate that T cells participate actively in liver injuries (Kim et al., 2015). Consequently, the LTT, which measures the specific drug T-cell response, has been employed to support the DILI diagnosis (Rotmensch et al., 1981; Takikawa et al., 2003; Pichler and Tilch, 2004; Kim et al., 2015; Whritenour et al., 2017; González‐Muñoz et al., 2020). The limitations of this test include the fact that the diagnostic performance depends on the clinical entity and drug involved in the adverse reaction and the lack of standardisation that contribute to the high variability between published studies regarding their accuracy (Mayorga et al., 2016; Mayorga et al., 2017). LTT shows an overall mean sensitivity and specificity of 56% and 94%, respectively, and it has been suggested that LTT has better diagnostic performance in moderate delayed drug hypersensitivity reactions than in severe reactions, such as organ-specific reactions (Mayorga et al., 2017). Low sensitivity is also observed in DILI. A large series of cases reported that LTT had a specificity of 100% and a sensitivity of 26% (Maria and Victorino, 1997). When cell cultures were performed in the presence of a prostaglandin inhibitor, the sensitivity increased to 56%, which would indicate the presence of suppressor cells in the cultures. Our results based on the classical LTT showed the same specificity (100%) but higher sensitivity in all patients (77%) and especially in the hepatocellular cases (92%). In the causality assessment, we used the RUCAM method that provides causality grading of suspected drugs and, unlike other causality assessment methods, includes the ruling out of alternative causes and individual scores and for all co-medications (Danan and Teschke, 2016; Yang et al., 2019). The use of different causality algorithms could account for the different sensitivity levels observed in LTT.

Given that a re-challenge test is contraindicated in DILI (Phillips et al., 2019), we explored two RUCAM scores in the ROC analysis as a true positive condition for DILI: ≥3 and ≥6. The SI threshold is the same for both analyses (SI ≥ 1.95); however, different sensitivity and specificity values were obtained. Greater differences could therefore be expected in the clinical performance of LTT when using distinct causality algorithms. Noticeably, the positivity threshold for SI obtained by the ROC curve analysis is equivalent to that arbitrarily considered in other studies (SI ≥ 2) (Maria and Victorino, 1997; Vílchez-Sánchez et al., 2020).

One of the striking results is that LTT showed a high sensitivity for the hepatocellular phenotype but moderate sensitivity for the cholestatic/mixed type. A possible reason for this could be that the cholestatic/mixed cases had a higher frequency of suppressor cells in the cultures that produced a negative LTT. However, there is no evidence supporting this possibility (Maria and Victorino, 1997). The difference in LTT sensitivity between the two phenotypes suggests that the adaptive immune response is more relevant in hepatocellular DILI than in cholestatic/mixed DILI and could be related to distinct pathogenic mechanisms operating under both DILI phenotypes (Kaplowitz, 2005; Andrade et al., 2019).

All DILI cases had a very short latency of 6 (4–16) days which is unusually short for idiosyncratic DILI and 75.5% exhibited only mild severity, which is also quite unusual. Through PPLSH, an early detection of DILI cases is carried out, 64.4% of the cases were developed during hospitalization at the beginning of DILI, being mild cases, except one moderate case, and with short latencies. However, 62.5% of the DILI cases that caused hospitalization were moderate or severe cases and with longer latencies. These data are consistent with previous DILI publications of the group (Pedraza et al., 2020; Delgado et al., 2021).

A strength of this study is the methodology of the prospective pharmacovigilance programme for detecting DILI cases, but there is the possibility that some DILI were missed during the process of attributing alternative causes through electronic medical records review (phase II). One limitation is that there are no controls for all drugs taken by the DILI cases. Nevertheless, the intention of including exposed controls was to analyse the performance of the test in a representative sample of controls. This has allowed to determine a general threshold that is often assumed arbitrary or is calculated with unexposed controls. Another limitation of this study is its small sample size and the results would need to be further validated in a large, prospective cohort. Nonetheless, our results demonstrate that LTT is an add on strengthening causality in cases of suspected idiosyncratic DILI, especially in patients with several suspected drugs and a hepatocellular phenotype.

## Data Availability

The raw data supporting the conclusion of this article will be made available by the authors upon reasonable request, without undue reservation.

## References

[B1] Aithal G. P. Watkins P. B. Andrade R. J. Larrey D. Molokhia M. Takikawa H. (2011). Case Definition and Phenotype Standardization in Drug-Induced Liver Injury. Clin. Pharmacol. Ther. 89 (6), 806–815. 10.1038/clpt.2011.58 21544079

[B2] Andrade R. J. Chalasani N. Björnsson E. S. Suzuki A. Kullak-Ublick G. A. Watkins P. B. (2019). Drug-induced Liver Injury. Nat. Rev. Dis. Primers 5 (1), 58. 10.1038/s41572-019-0105-0 31439850

[B3] Danan G. Teschke R. (2019). Roussel Uclaf Causality Assessment Method for Drug-Induced Liver Injury: Present and Future. Front. Pharmacol. 10, 853–858. 10.3389/fphar.2019.00853 31417407 PMC6680600

[B4] Danan G. Teschke R. (2016). RUCAM in Drug and Herb Induced Liver Injury: The Update. Int. J. Mol. Sci. 17, 14. 10.3390/ijms17010014 PMC4730261 26712744

[B5] Delgado A. Stewart S. Urroz M. Rodríguez A. Borobia A. M. Akatbach-Bousaid I. (2021). Characterisation of Drug-Induced Liver Injury in Patients with COVID-19 Detected by a Proactive Pharmacovigilance Program from Laboratory Signals. J. Clin. Med. 10 (19), 4432. 10.3390/jcm10194432 34640458 PMC8509270

[B6] García-Cortés M. Stephens C. Lucena M. I. Fernández-Castañer A. Andrade R. J. (2011). Causality Assessment Methods in Drug Induced Liver Injury: Strengths and Weaknesses. J. Hepatol. 55 (3), 683–691. 10.1016/j.jhep.2011.02.007 21349301

[B7] González‐Muñoz M. Monserrat Villatoro J. Marín‐Serrano E. Stewart S. Bardón Rivera B. Marín J. (2020). A Case Report of a Drug‐induced Liver Injury (DILI) Caused by Multiple Antidepressants with Causality Established by the Updated Roussel Uclaf Causality Assessment Method (RUCAM) and *In Vitro* Testing. Clin. Case Rep. 8 (12), 3104–3108. 10.1002/ccr3.3348 PMC7752543 33363890

[B8] Kaplowitz N. (2005). Idiosyncratic Drug Hepatotoxicity. Nat. Rev. Drug Discov. 4 (6), 489–499. 10.1038/nrd1750 15931258

[B9] Kim S. H. Saide K. Farrell J. Faulkner L. Tailor A. Ogese M. (2015). Characterization of Amoxicillin- and Clavulanic Acid-specific T Cells in Patients with Amoxicillin-Clavulanate-Induced Liver Injury. Hepatology 62 (3), 887–899. 10.1002/hep.27912 25998949

[B10] Maria V. A. Victorino R. M. (1997). Diagnostic Value of Specific T Cell Reactivity to Drugs in 95 Cases of Drug Induced Liver Injury. Gut 41 (4), 534–540. 10.1136/gut.41.4.534 9391255 PMC1891538

[B11] Mayorga C. Celik G. Rouzaire P. Whitaker P. Bonadonna P. Rodrigues-Cernadas J. (2016). *In Vitro* tests for Drug Hypersensitivity Reactions: an ENDA/EAACI Drug Allergy Interest Group Position Paper. Allergy 71 (8), 1103–1134. 10.1111/all.12886 26991315

[B12] Mayorga C. Doña I. Perez-Inestrosa E. Fernández T. D. Torres M. J. (2017). The Value of *In Vitro* Tests to DiminishDrug Challenges. Int. J. Mol. Sci. 18 (6), 1222. 10.3390/ijms18061222 PMC5486045 28590437

[B13] Pedraza L. Laosa O. Rodríguez-Mañas L. Gutiérrez-Romero D. F. Frías J. Carnicero J. A. (2020). Drug Induced Liver Injury in Geriatric Patients Detected by a Two-Hospital Prospective Pharmacovigilance Program: A Comprehensive Analysis Using the Roussel Uclaf Causality Assessment Method. Front. Pharmacol. 11, 600255. 10.3389/fphar.2020.600255 33613279 PMC7892439

[B14] Phillips E. J. Bigliardi P. Bircher A. J. Broyles A. Chang Y. S. Chung W. H. (2019). Controversies in Drug Allergy: Testing for Delayed Reactions. J. Allergy Clin. Immunol. 143 (1), 66–73. 10.1016/j.jaci.2018.10.030 30573342 PMC6429556

[B15] Pichler W. J. Tilch J. (2004). The Lymphocyte Transformation Test in the Diagnosis of Drug Hypersensitivity. Allergy 59 (8), 809–820. 10.1111/j.1398-9995.2004.00547.x 15230812

[B16] Ramirez E. Carcas A. J. Borobia A. M. Lei S. H. Piñana E. Fudio S. (2010). A Pharmacovigilance Program from Laboratory Signals for the Detection and Reporting of Serious Adverse Drug Reactions in Hospitalized Patients. Clin. Pharmacol. Ther. 87 (1), 74–86. 10.1038/clpt.2009.185 19890254

[B17] Rotmensch H. H. Leiser A. Dan M. Klejman A. Livni E. Ilie B. (1981). Evaluation of Prajmalium-Induced Cholestasis by Immunologic Tests. Arch. Intern. Med. 141 (13), 1797–1801. 10.1001/archinte.141.13.1797 7316626

[B18] Takikawa H. Takamori Y. Kumagi T. Onji M. Watanabe M. Shibuya A. (2003). Assessment of 287 Japanese Cases of Drug Induced Liver Injury by the Diagnostic Scale of the International Consensus Meeting. Hepatol. Res. 27 (3), 192–195. 10.1016/s1386-6346(03)00232-8 14585395

[B19] Teschke R. Danan G. (2020). Worldwide Use of RUCAM for Causality Assessment in 81,856 Idiosyncratic DILI and 14,029 HILI Cases Published 1993-Mid 2020: A Comprehensive Analysis. Medicines (Basel) 7 (10), 62. 10.3390/medicines7100062 PMC7600114 33003400

[B20] Teschke R. (2019). Idiosyncratic DILI: Analysis of 46,266 Cases Assessed for Causality by RUCAM and Published from 2014 to Early 2019. Front. Pharmacol. 10, 1–24. 10.3389/fphar.2019.00730 31396080 PMC6664244

[B21] Vílchez-Sánchez F. Loli-Ausejo D. Rodriguez-Mariblanca A. Montserrat-Villatoro J. Ramírez E. Domínguez-Ortega J. (2020). Lymphocyte Transformation Test Can Be Useful for the Diagnosis of Delayed Adverse Reactions to Sulfonamides. Allergy 75 (12), 3267–3272. 10.1111/all.14437 32506576

[B22] Weber S. Benesic A. Neumann J. Gerbes A. L. (2021). Liver Injury Associated with Metamizole Exposure: Features of an Underestimated Adverse Event. Drug Saf. 44 (6), 669–680. 10.1007/s40264-021-01049-z 33638811 PMC8184550

[B23] Whritenour J. Ko M. Zong Q. Wang J. Tartaro K. Schneider P. (2017). Development of a Modified Lymphocyte Transformation Test for Diagnosing Drug-Induced Liver Injury Associated with an Adaptive Immune Response. J. Immunotoxicol 14 (1), 31–38. 10.1080/1547691X.2016.1254305 28121193 PMC5505862

[B24] Wuillemin N. Terracciano L. Beltraminelli H. Schlapbach C. Fontana S. Krähenbühl S. (2014). T Cells Infiltrate the Liver and Kill Hepatocytes in HLA-B(∗)57:01-associated Floxacillin-Induced Liver Injury. Am. J. Pathol. 184 (6), 1677–1682. 10.1016/j.ajpath.2014.02.018 24731753

[B25] Yang H. Guo D. Xu Y. Zhu M. Yao C. Chen C. (2019). Comparison of Different Liver Test Thresholds for Drug-Induced Liver Injury: Updated RUCAM versus Other Methods. Front. Pharmacol. 10, 816. 10.3389/fphar.2019.00816 31379581 PMC6658872

